# *Peg3* Deficiency Results in Sexually Dimorphic Losses and Gains in the Normal Repertoire of Placental Hormones

**DOI:** 10.3389/fcell.2018.00123

**Published:** 2018-09-27

**Authors:** Simon J. Tunster, Raquel Boqué-Sastre, Gráinne I. McNamara, Susan M. Hunter, Hugo D. J. Creeth, Rosalind M. John

**Affiliations:** Biomedicine Division, School of Biosciences, Cardiff University, Cardiff, United Kingdom

**Keywords:** *Peg3*, placenta, genomic imprinting, sexually dimorphic phenotype, placental hormones

## Abstract

Hormones from the fetally derived placenta signal to the mother throughout pregnancy to ensure optimal fetal growth and prepare the mother for her new role in nurturing her offspring. Through evolution, placental hormones have under gone remarkable diversification and species-specific expansions thought to be due to constant rebalancing of resource allocation between mother and offspring. Genomic imprinting, an epigenetic process in which parental germlines silence genes in the offspring, is thought to be the physical embodiment of a second conflicting interest, between the male and female mammal. Several genes silenced by paternal imprints normally function to limit the placental endocrine lineages of the mouse placenta. We hypothesized that paternal imprinting has adapted to overcome the rapid evolution of placental hormone gene families by directly regulating the lineages that express these hormones rather than individual hormones. This predicts the existence of genes maternally silenced in the offspring counteracting the influence of the paternal imprint. Here we report on the consequences of loss of function of *Paternally expressed gene 3* (*Peg3*), on placental endocrine lineages. Mutant male placenta displayed a marked loss of the spongiotrophoblast, a key endocrine lineage of the placenta, and the glycogen cell lineage alongside reduced stores of placental glycogen and changes in expression of the normal repertoire of placental hormones. *Peg3* is known to transcriptionally repress placental hormone genes. *Peg3* consequently both positively and negatively regulates placental hormones through two independent and opposing mechanisms. Female placenta showed moderate response to loss of *Peg3* with minor alterations to the junctional zone lineages and few changes in gene expression. These data highlight the important fact that female placenta compensate for the loss of *Peg3* better than male placenta. This work lends further support to our novel hypothesis that the parental genomes are competing over the endocrine function of the mouse placenta and further suggests that a conflict between males and females begins *in utero*.

## Introduction

Eutherian mammals provide nutrients to their young *in utero* via the fetally derived placenta enabling prolonged gestation and the birth of relatively mature offspring central to their reproductive success (John and Hemberger, 2012). Across mammalian species there are a wide variety of placental shapes, sizes, and structures (Carter and Enders, 2004; Capellini et al., 2011) as well as an extensive diversification of several placental hormone gene families (Rawn and Cross, 2008). These rapid changes are thought to reflect the antagonistic yet interdependent relationship between mother and offspring with higher growth rates favoring the offspring and counter-adaptation by the mother to preserve her future reproductive potential (Haig, 1996). A second antagonistic relationship exists between males and females as a consequence of the inequality of resource provision to their mutual offspring. Genomic imprinting, an epigenetic process whereby the paternal and maternal germlines have switched off certain genes inherited by their offspring (Surani, 1998), is thought to be a result of these conflicting interests (Moore and Haig, 1991). However, the continual evolution of placental hormones, which act to extract resources from the mother, poses a unique challenge for this theory of genomic imprinting as rapid evolution would allow genes to continually escape direct imprinting. We previously reported on three maternally silenced genes that function to restrain the development of endocrine lineages of the mouse placenta (Tunster et al., 2010, 2011, 2014, 2016a,b). This led us to hypothesize that the paternal genome has overcome the rapid evolution of placental hormone genes by regulating placental endocrine lineages rather than specific genes. However, evidence for maternal silencing of genes to counteract the action of the paternal genome providing more compelling support for this hypothesis is lacking.

In mice, seven distinct and identifiable lineages manufacture placental hormones (Simmons et al., 2007, 2008; Gasperowicz et al., 2013; Rai and Cross, 2014). The spongiotrophoblast is the most substantial endocrine lineage in terms of cell number (Coan et al., 2006), forming the bulk of the junctional zone sandwiched between the decidua (maternal component of the placenta) and the labyrinth (fetally derived, nutrient exchange). Paternally silenced *Pleckstrin Homology-Like Domain, Family A, Member 2* (*Phlda2*) functions to specifically to restrict the expansion of this lineage with just twofold increased expression reducing the contribution of the spongiotrophoblast to the mature placenta by ∼50% (Tunster et al., 2010, 2016a). Conversely, loss-of-expression of *Phlda2* results in a twofold expansion, co-incident with changes in the expression of spongiotrophoblast-expressed hormones (Tunster et al., 2016a). Paternally silenced *Achaete-scute complex homolog 2* (*Ascl2* aka *Mash2*) also functions to restrict the expansion of the spongiotrophoblast (Tunster et al., 2016b) while paternally silenced *Cyclin dependent kinase inhibitor 1c* (*Cdkn1c*) is required for the proper development of this lineage (Tunster et al., 2011). *Cdkn1c* support the differentiation of the second major cell type of the junctional zone, the glycogen cell lineage, recognized by stores of glycogen that accumulate as gestation proceeds (Coan et al., 2006). Additionally, there are a number of other genes paternally silenced by virtue of their location on the paternally inactivated X chromosome that regulate cell types within the junctional zone (John and Hemberger, 2012). The spongiotrophoblast and glycogen cell lineages expresses members of the *placental lactogen* gene family (*Prls*) (Simmons et al., 2007, 2008). *Prls* are related to the pituitary hormone prolactin, and some members of this family play an important role in driving the maternal adaptations required for a successful pregnancy (Muller et al., 1999; Bhattacharyya et al., 2002). There are 22 Prl family members in mice of which only Prl3d1 (PL-I) and Prl3b1 (PL-II) have formally been shown to signal via the prolactin receptor (Soares et al., 2007). In addition to *Prls*, the spongiotrophoblast lineage expresses *pregnancy specific glycoproteins* (*Psgs*), a 17 member multigene gene family that contributes to the protection of the semiallotypic fetus from the maternal immune system and remodels placental and maternal vasculature (Moore and Dveksler, 2014). The spongiotrophoblast lineage is important for fetal growth (Tunster et al., 2010, 2014, 2016a) and the priming of maternal care during pregnancy (Creeth et al., 2018), which place demands on maternal resources.

Five distinct trophoblast giant cell (TGC) lineages, recognized by their giant nuclei, also express *Prls* (Simmons et al., 2007). The secondary parietal (P-) TGC are located as a single discontinuous layer of cells between the maternal decidua, and junctional zone (Simmons et al., 2007). Overexpression of *Ascl2* results 40% fewer P-TGCs (Tunster et al., 2016b). Sinusoidal TGCs (S-; previously called trophoblast layer I) are in direct contact with maternal blood spaces in the labyrinth (Simmons et al., 2007, 2008) and *Cdkn1c* is required for their proper development (Tunster et al., 2011). Spiral artery (SpA-) TGCs line the maternal blood system in the maternal decidua before entry into the junctional zone, canal (C-) TGCs line the maternal blood canals entering the junctional zone and passing through to the labyrinth, and channel (Ch-) TGCs line the maternal blood spaces located beneath the decidua where maternal blood leaves the placenta (Gasperowicz et al., 2013; Rai and Cross, 2014). Alterations in the expression of *Phlda2*, *Ascl2*, and *Cdkn1c* do not appear to have a major impact on these lineages. Only one maternally silenced gene, *insulin-like growth factor 2* gene (*Igf2*), has been well characterized with respect to the placental endocrine lineages. Gain-in-expression of *Igf2* promotes the expansion of the glycogen cell and P-TGC lineages with minimal effect on the spongiotrophoblast (Lopez et al., 1996; Esquiliano et al., 2009).

The function of *Phlda2*, *Ascl2*, and *Cdkn1c* in regulating the spongiotrophoblast calls attention to this lineage as site of action for genomic imprinting. However, a paternally expressed gene with an antagonist function on this lineage has not been reported. The maternally silenced *Paternally expressed gene 3* (*Peg3*) plays an important role in both fetal and placental development (Li et al., 1999; Curley et al., 2004). Altered expression of a number of spongiotrophoblast-expressed genes in *Peg3* knock out (KO) placenta (Broad et al., 2009; Kim et al., 2013) suggests changes in the relative contribution of this lineage to the placenta. However, not all the reported gene changes were consistent with a simple loss or gain of one or more endocrine lineages. Transcriptional studies on *Peg3* are complicated by its function as a zinc finger protein silencing the expression of a number of placental hormone genes, including several *Prls* and *Psgs* (Broad and Keverne, 2011; Thiaville et al., 2013; Lee et al., 2015) that are used as markers to identify the seven placental endocrine lineages. Consequently changes in their expression could indicate either a change in cell number or changes in expression within a specific cell type, or potentially both outcomes, highlighting one of the challenges of using cell lineage markers on heterogeneous tissues. To overcome this challenge, we employed a combination of classic histology and gene expression profiling with the newly developed RNAscope technology to characterize placental lineages in male and female *Peg3* KO placenta. In addition to finding support for our original hypothesis of parental conflict over the spongiotrophoblast, we observed differences in both the placental lineages and gene transcription between male and female *Peg3* KO placenta.

## Results

**FIGURE 1:**
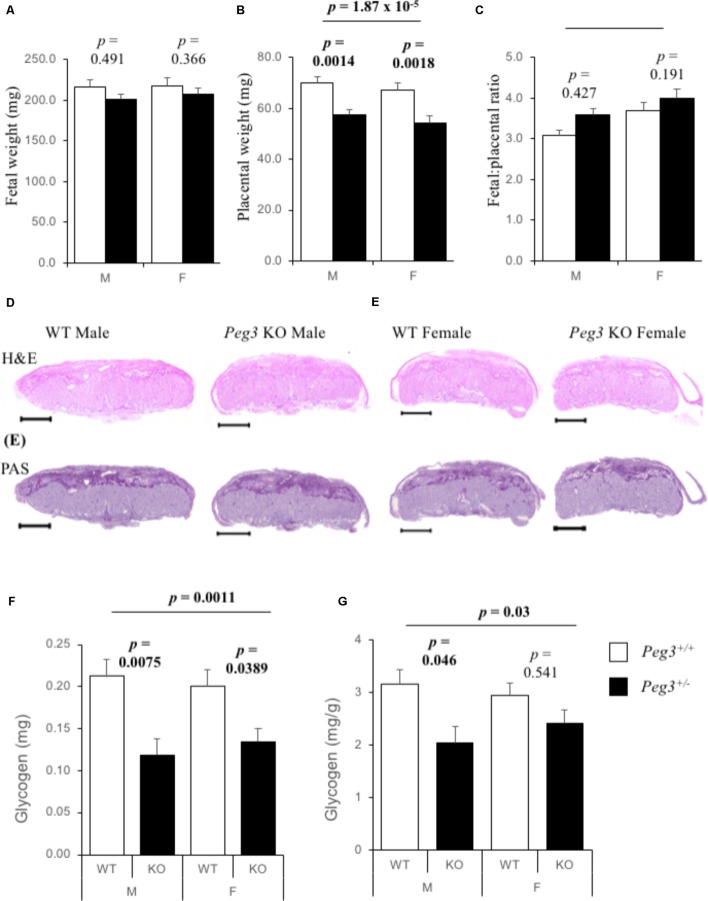
Reduction in placental glycogen stores in *Peg3* deficient placenta. **(A)** Wet weights of male and female WT and *Peg3* KO fetuses at E14.5. No difference between WT male (*N* = 22), and WT female (*N* = 24), fetal weights, or between WT male and *Peg3* KO male (*N* = 22), or between WT female and *Peg3* KO female (*N* = 20), fetal weights. Data from 11 litters, litter size between 5 and 10. **(B)** Wet weights of WT and *Peg3* KO placenta at E14.5. No difference in weight between WT male and WT female placenta. Male and female *Peg3* KO placenta weighed 18 and 19% less than sex-matched WT placenta. **(C)** Fetal:Placental (F:P) ratios at E14.5. **(D)** Hematoxylin and eosin staining of male and female WT, and *Peg3* KO midline placental sections at E14.5. **(E)** PAS staining of adjacent male and female WT and *Peg3* KO midline placental sections. **(F)** Direct biochemical determination of total glycogen (mg) present in male and female WT and *Peg3* KO placenta at E14.5. Male WT placenta contain same total glycogen as female placenta (*p* = 0.69). Male and female *Peg3* KO placenta contain 46 and 33% less total placental glycogen than sex-matched WT placenta. **(G)** Glycogen expressed as mg/g placenta. Male and female *Peg3* KO placenta contain 35 and 18% less placental glycogen per gram than sex-matched WT placenta. Scale bars = 1000 μm. Error bars represent SEM. Statistical significance calculated from ANOVA with Bonferroni correction. Data in **Supplementary Table S1**.

We bred *Peg3*^-/+^ (maternal inheritance of targeted allele) *Mus musculus* 129 mouse strain males derived from the original line (Li et al., 1999; Curley et al., 2004) with wild type (WT) 129 females to generate *Peg3*^+/-^ (paternal inheritance of targeted allele; KO) and matched *Peg3*^+/+^ (WT) fetuses and placenta at E14.5. There were no differences in fetal weights at E14.5 between WT and *Peg3* KO fetuses, and no difference by fetal sex (**Figure 1A**). *Peg3* KO placenta were 18–20% lighter than their WT counterparts and wet weights did not differ by fetal sex (**Figure 1B**). Fetal:placental ratios were not significantly different (**Figure 1C**). Hematoxylin and eosin (H&E) staining of male and female WT and KO sections suggested subtle differences in the junctional zone with less cell free space (**Figure 1D**). The intensity of Periodic Acid Schiff (PAS) staining, which identifies glycogen stores (Coan et al., 2006), was similar between the four genotypes (**Figure 1E**). A biochemical quantification of placental glycogen revealed a substantial difference in total glycogen (*F*_3,81_ = 5.88, *p* = 0.00110; **Figure 1F**). Male *Peg3* KO placenta possessed 43% less total glycogen (*p***=** 0.0075) and female *Peg3* KO placenta possessed 35% less (*p* = 0.0389). Glycogen was different as a proportion of placental weight (*F*_3,81_ = 5.88, *p* = 0.03) but only between WT and KO male placenta (*p* = 0.046; **Figure 1G**).

**FIGURE 2:**
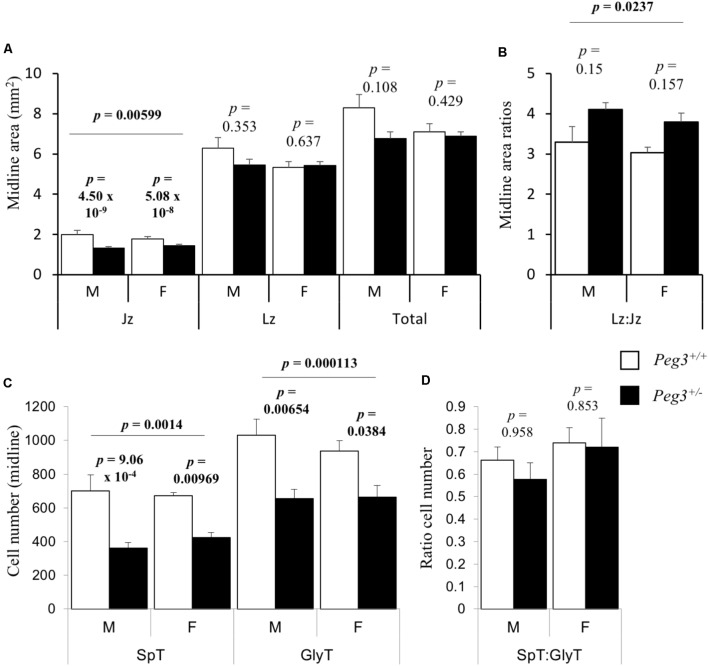
Sexually dimorphic loss of spongiotrophoblast and glycogen cells from junctional zone in response to loss of *Peg3*. **(A)** Junctional zone (Jz), labyrinth zone (Lz), and total areas of midline E14.5 placental sections (mm^2^) from WT, and *Peg3* KO male and female placenta. **(B)** Labyrinth to junctional zone midline area ratios. **(C)** Spongiotrophoblast (Spt) and glycogen cell (GlyT) number cell number within midline areas of the junctional zone**. (D)** Ratio of spongiotrophoblast to glycogen cell number within the junctional zone. *N* = 6–7 placenta per genotype, sex matched within litter. Seven independent litters; error bars represent SEM. Statistical significance calculated from ANOVA with Bonferroni correction. Data in **Supplementary Table S2**.

A reduction in placental glycogen stores can reflect fewer glycogen cells, as reported in placenta lacking the paternally expressed *Igf2* (Lopez et al., 1996). Alternatively, we have observed a reduction in placental glycogen when just the spongiotrophoblast lineage is reduced (Tunster et al., 2016a). Gross area measurements of midline sections from seven placenta of each sex/genotype revealed that junctional zone areas were different between the groups (*F*_3,24_ = 5.83, *p* = 0.00599). *Peg3* KO male junctional zone midline area was 34% smaller than WT male placenta (*p* = 4.50 × 10^-9^; **Figure 2A**). Female *Peg3* KO placenta showed a more modest 19% reduction in the junctional zone midline area compared to WT female placenta (*p* = 5.08 × 10^-8^; **Figure 2A**). The increase in Labyrinth zone:Junctional zone (Lz:Jz) ratio between WT and KO placenta was not significant (**Figure 2B**). Quantitative analysis of junctional zone cell numbers from mid line sections revealed a difference in the number of spongiotrophoblast (*F*_3,24_ = 10.3, *p* = 0.000113) and glycogen cells (*F*_3,24_ = 7.12, *p* = 0.0014) (**Figure 2C**). Male *Peg3* KO placenta possessed 50% fewer spongiotrophoblast cells (*p* = 9.06 × 10^-4^), and female *Peg3* KO placenta showed an attenuated 37% fewer spongiotrophoblast cells (*p* = 0.00969). Male *Peg3* KO placenta possessed 40% fewer glycogen cells than WT male placenta (*p* = 0.00654), and female *Peg3* KO placenta possessed 30% fewer glycogen cells than WT female controls (*p* = 0.0384). Male and female *Peg3* KO placenta retained a normal ratio of spongiotrophoblast to glycogen cells (**Figure 2D**).

Between WT male and female placenta from *Peg3* KO litters, the majority of markers of the spongiotrophoblast, TGC lineages, and labyrinth were expressed at similar levels (**Supplementary Figure S1** and **Supplementary Table S1**). *Trophoblast specific protein alpha* [*Tpbpa;* expressed in glycogen cells, spongiotrophoblast, SpA-TGCs, C-TGCs, and 50% of P-TGCs (Hu and Cross, 2011)], *Cdkn1c* [glycogen cells, P-TGCs and S-TGCs (Riley et al., 1998; Westbury et al., 2001; Georgiades et al., 2002; Coan et al., 2006)], *Prl2a1* [glycogen cells, SpA-TGCs and C-TGCs (Simmons et al., 2008)], and exclusive markers of the glycogen cell lineage *Protocadherin 12* (*Pcdh12*) (Bouillot et al., 2006), *1,4-alpha-glucan-branching enzyme* (*Gbe1*), and *UDP-glucose pyrophosphorylase 2* (*Ugp2*) were all expressed at higher levels in male placenta at E14.5 (**Supplementary Figure S1**). This did not translate as increased total placental glycogen (0.212 mg ± 0.02 vs. 0.201 mg ± 0.02; *P* = 0.69), or glycogen per mg of placental weight (3.16 mg/g ± 0.28 vs. 2.94 mg/g ± 0.25; *P* = 0.57), or a higher glycogen cell number (1031 ± 95.9 vs. 937 ± 54.6; *P* = 0.42), in WT male placenta, at least at E14.5 (**Supplementary Tables S1**, **S2**).

**FIGURE 3:**
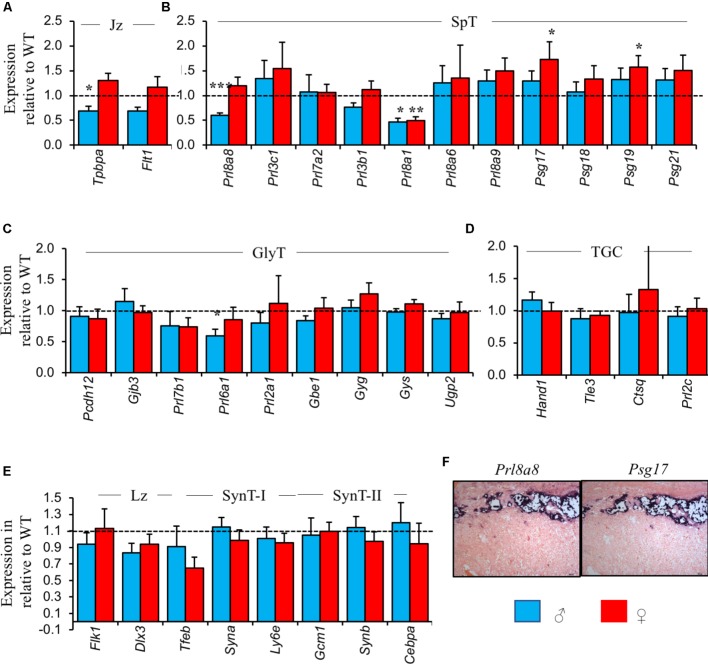
Placental lineage analysis by RT-qPCR reveals differences in the normal repertoire of placental hormone gene expression between male and female *Peg3* KO placenta. **(A)** RT-qPCR comparison of mature junctional zone markers *Tpbpa* and *Flt1* at E14.5 in male and female *Peg3* KO placenta relative to sex-matched WT placenta. **(B)** RT-qPCR comparison of markers exclusively or predominantly expressed in the mature spongiotrophoblast lineage. **(C)** RT-qPCR comparison of markers exclusively or predominantly expressed in the mature glycogen cell lineage. **(D)** RT-qPCR comparison of pan-TGC markers. **(E)** RT-qPCR comparison of markers of the mature labyrinth, syncytiotrophoblast layers I and II (SynT-I and SynT-II). **(F)** *In situ* hybridization of adjacent E14.5 placental sections with *Prl8a8* and *Psg17* riboprobes. Scale bars = 100 mm. For the RT-qPCR analysis, *N* = 4 placenta per genotype and sex, total *N* = 16 (2 vs. 2 from 2 independent litters, and 2 vs. 2 of each sex); error bars represent SEM. Statistical significance calculated from ANOVA with Bonferroni correction. ^∗^*P* < 0.05. Data in **Supplementary Table S3** and at https://osf.io/jhc83/.

Between WT and *Peg3* KO male placenta, reduced expression of markers of the spongiotrophoblast and glycogen cell lineages was consistent with the loss of both these cells types determined histologically. *Tpbpa* was 30–40% lower in *Peg3* KO male placenta relative to WT male controls (**Figure 3A**). *Prl8a8* and *Prl8a1*, two markers predominantly or exclusively expressed in the spongiotrophoblast (Simmons et al., 2008; **Figure 3B**) and *Prl6a1*, an exclusive marker for glycogen cells (Simmons et al., 2008) were similarly reduced in expression (**Figure 3C**) while genes expressed in the labyrinth and pan markers of TGCs were expressed at WT levels (**Figures 3D,E**). Not all genes expressed from the spongiotrophoblast and glycogen cell lineages showed reduced expression in male *Peg3* KO placenta. Strikingly, few significant reductions in gene changes were apparent in female *Peg3* KO placenta and *Psg17* and *Psg19*, co-expressed with *Prl8a8* in spongiotrophoblast (Wynne et al., 2006; **Figure 3F**), were expressed at significantly higher levels than WT despite the 37% loss of this lineage.

Previously, β-galactosidase staining revealed broad expression of *Peg3* in the junctional zone, within cells with large nuclei lining the region between the junctional zone and maternal decidua identified as P-TGCs, and some unidentified cells in the labyrinth (Hiby et al., 2001). We asked whether RNAscope, an ultrasensitive *in situ* hybridization amplification technique (Wang et al., 2012), could provide a more sensitive approach for identifying cell types expressing *Peg3*. This approach allows the detection of individual target RNAs within intact cells in alongside multiplex analysis for multiple markers which can aid lineage identification. We first applied a commercially available *Peg3* RNAscope probe (**Supplementary Figure S2A**), to WT paraformaldehyde-fixed sections which revealed a strong punctuate signal in the nucleus with weaker staining within the cytoplasm (**Supplementary Figures S2B,C**). An identical localization was apparent with a custom designed *Peg3* probe spanning a different region of *Peg3* (**Supplementary Figures S2D,E**).

**FIGURE 4:**
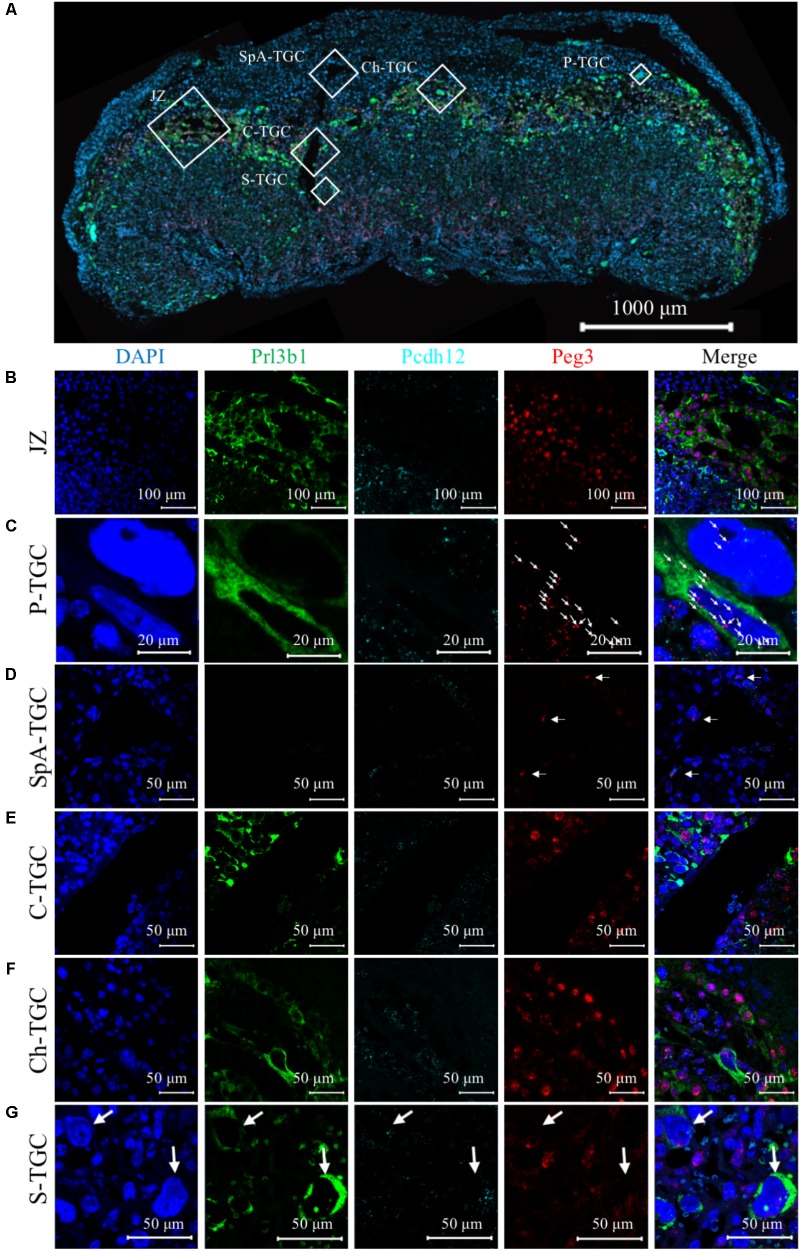
Three-plex detection of *Peg3*, *Pcdh12*, and *Prl3b1* identifies *Peg3*+ve cells. **(A)** Low magnification image of whole E14.5 female WT placental section after *in situ* amplification of *Peg3* (red), *Pcdh12* (turquoise), and *Prl3b1* (*Pl2*; green). Nuclei labeled with DAPI (blue). **(B)** Higher resolution image of region indicated by open white box labeled Jz in 4A. *Peg3* signal localizes to both *Prl3b1*+ve spongiotrophoblast cells and *Pcdh12*+ve glycogen cells. Further images in **Supplementary Figure S3**. **(C)** Higher resolution image of region indicated by open white box labeled P-TGC in panel **(A)**. Single RNA molecule detection of *Peg3* localized to large *Prl3b1*+ve mononuclear cells lining region between decidua and junctional zone indicated by white arrows. **(D)** Higher resolution image of region indicated by open white box labeled SpA-TGC in panel **(A)**. *Peg3* signal in *Prl3b1*-ve/*Pcdh12*+ve cells surrounding maternal spiral artery within decidua indicated by white arrows. **(E)** Higher resolution image of region indicated by open white box labeled C-TGC in panel **(A)**. *Peg3* signal in *Prl3b1*+ve cells lining wide channels where maternal blood enters the labyrinth. **(F)** Higher resolution image of region indicated by white boxed region labeled Ch-TGC in panel **(A)**. *Peg3* signal in *Prl3b1*+ve cells lining small channels within the junctional zone. **(G)** Higher resolution image of region indicated by white boxed region labeled S-TGC in panel **(A)**. *Peg3* signal in some large *Prl3b1*+ve mononuclear cells located lining the maternal blood sinusoids indicated by white arrows and some *Prl3b1*-ve cells with small nuclei in labyrinth.

We performed triple detection of *Peg3* (red) with *Prl3b1* (green) to identify spongiotrophoblast cells, P-TGCs, C-TGCs, and S-TGCs (Simmons et al., 2008), and *Pcdh12* (turquoise), to identify glycogen cells (Bouillot et al., 2006; **Figure 4A**). *Peg3* was expressed in both *Prl3b1*+ve, and *Pcdh12*+ve cells within the junctional zone (**Figure 4B**). *Peg3* signal within the *Prl3b1*+ve spongiotrophoblast cells was stronger, and more nuclear in comparison to the neighboring *Pcdh12*+ve glycogen cells. Some cells with large nuclei located at the periphery of the junctional zone adjacent to the decidua and strongly positive for *Prl3b1*, indicative of P-TGC cells, were weakly positive for *Peg3* signal (**Figure 4C** and **Supplementary Figure S3**). A few cells adjacent to the spiral arteries within the decidua were positive for *Peg3* and *Pcdh12* identifying migrated glycogen cells, not SpA-TGCs (**Figure 4D**). *Peg3* signal was apparent in *Prl3b1*+ve cells with large nuclei lining the maternal vasculature on entry into the labyrinth (Canal TGCs; **Figure 4E**) and exiting via junctional zone (Channel TGCs) (**Figure 4F**). *Peg3* was co-expressed with *Prl3b1* in some cells in the labyrinth indicative of S-TGCs (indicated by arrow **Figure 4G**). In addition, there were some cells with small nuclei (indicated by asterisks) that were *Prl3b1-ve/Peg*3+ve, and large cells that were *Prl3b1+ve/Peg*3-ve (**Figure 4G**). This reciprocal relationship between *Prl3b1* and *Peg3* was consistent with the role of Peg3 transcriptionally repressing *Prl3b1* expression (Kim et al., 2013). In summary, *Peg3* was expressed in all endocrine lineages except the SpA-TGCs.

**FIGURE 5:**
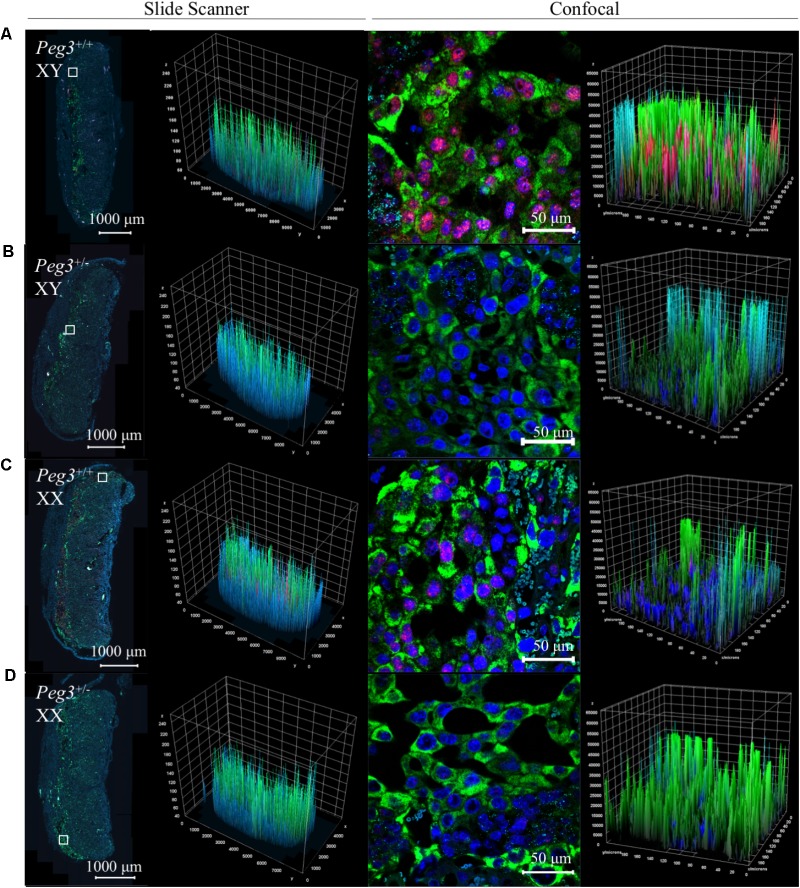
Three-plex detection of *Peg3*, *Pcdh12*, and *Prl3b1* expression in WT and *Peg3*KO male and female E14.5 placenta. Images of whole E14.5 placental sectional and junctional zone after *in situ* amplification of *Peg3* (red), *Pcdh12* (turquoise), and *Prl3b1* (*Pl2*; green). Nuclei labeled with DAPI (blue). All placenta are from the same litter and treated in an identical manner to generate the images. **(A)** WT male placenta. **(B)** Peg3 KO male placenta. **(C)** WT female placenta. **(D)** Peg3 KO female placenta. Scale bars between confocal and slide scanner are different because the confocal images at 16 bits and the slide scanner images at 8 bits. Z = fluorescence intensity value; Y and X = area of the sample.

Triple detection of *Peg3*, *Pcdh12*, and *Prl3b1* on placental samples from a single litter further illustrated the differences between WT and *Peg3* KO sections from male and female placenta identified histologically (**Figures 5A–D**). The male *Peg3* KO placenta (**Figure 5B**) had relatively fewer *Prl3b1*+ve cells compared to the male WT placenta (**Figure 5A**) and the female *Peg3* KO placenta (**Figure 5D**) expressed *Prl3b1* at higher levels compared to the matched female WT placenta (**Figure 5C**). Although assessed qualitatively, these data highlight the future potential for this approach in the detailed characterization of placental lineages.

**FIGURE 6:**
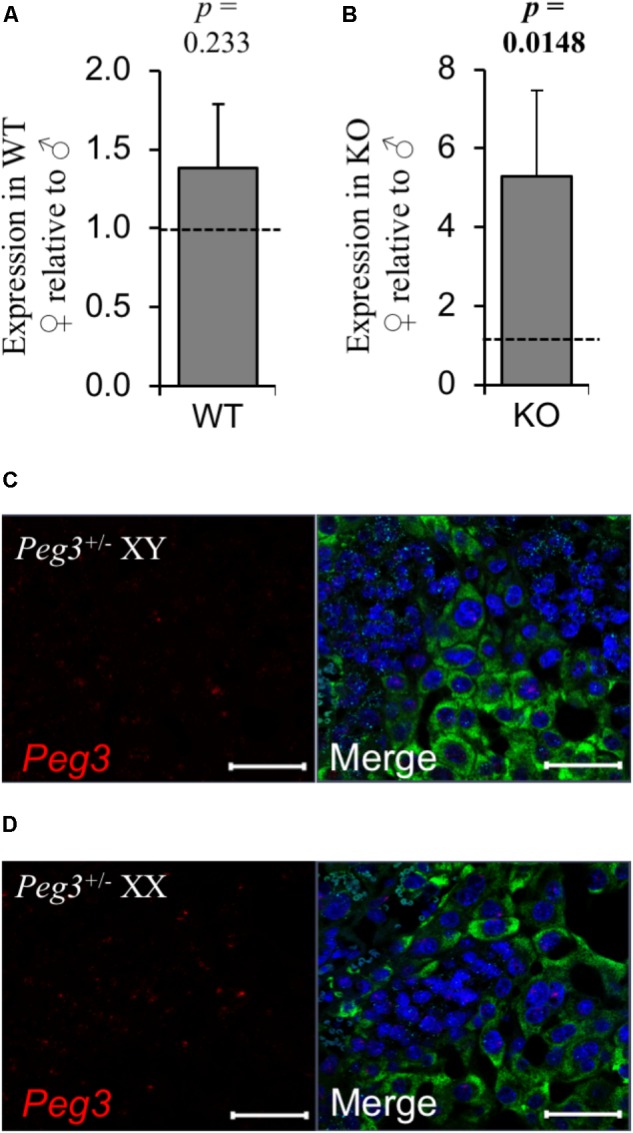
No evidence for substantial relaxation of maternal silencing in female placenta. **(A)** RT-qPCR comparison of *Peg3* expression between WT male and WT female placenta at E14.5. **(B)** RT-qPCR comparison of *Peg3* expression between KO male and KO female placenta at E14.5. **(C,D)** High resolution image of three-plex analysis of junctional zone of male and female placenta inheriting the targeted Peg3 allele maternally (silenced). For the RT-qPCR analysis, *N* = 4 placenta per genotype (2 vs. 2 from 2 independent litters); error bars represent SEM. Statistical significance calculated using from ANOVA with Bonferroni correction. Data in **Supplementary Table S4**.

Both male and female placenta were impacted by loss-of-function of *Peg3* but female placenta presented with a considerably more subtle phenotype. Sexually dimorphic expression has been reported for *Peg3* (Faisal et al., 2014) but on the 129 strain background there was no difference in the expression of *Peg3* between E14.5 WT male and female placenta (**Figure 6A**). *Peg3* expression was fivefold higher in female *Peg3* KO placenta compared to male *Peg3* KO placenta (**Figure 6B**). This was at considerably lower that the normal level of expression in WT female placenta (**Supplementary Table S4**) illustrated by RNAscope analysis of the junctional zone after maternal transmission of the targeted allele (**Figures 6C,D**).

## Discussion

**FIGURE 7:**
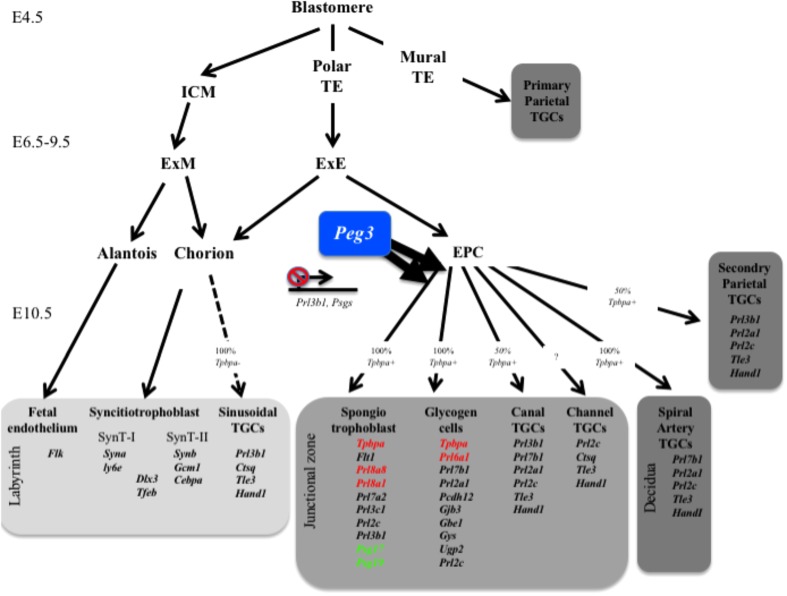
Summary of gene expression data in relation to placental lineages. *Peg3* is required for the expansion of both the glycogen cell and spongiotrophoblast lineages. Genes indicated in red are significantly reduced in expression in male *Peg3* KO placenta. Genes indicated in green are significantly elevated in female *Peg3* KO placenta. ICM, inner cell mass; TE, trophectoderm; TGCs, trophoblast giant cells; ExM, extraembryonic mesoderm; ExE, extraembryonic ectoderm; EPC, ectoplacental cone; SynT, syncitiotrophoblast.

We previously reported that several paternally silenced imprinted genes converge on the placental endocrine lineages to limit the production of placental hormones. This led us to hypothesize that the male genome adapted to overcome the rapid evolution of placental hormone gene families by directly regulating the lineages that express placental hormones rather than individual hormone genes. Here, we demonstrate that the maternally silenced *Peg3* gene is required for the expansion of the spongiotrophoblast and glycogen cell lineages consistent with our original hypothesis (**Figure 7**), with additional intriguing complexities.

### *Peg3* Positively and Negatively Regulates Placental Hormones

This study applied both classic histological techniques and a cutting-edge imaging approach to explore the phenotypic consequences of loss of *Peg3* function in the mouse placenta in more detail. Male *Peg3* KO placenta showed a 50% loss of spongiotrophoblast, a 40% loss of glycogen cells, and a 45% reduction in placental glycogen alongside 40–60% reduction in expression of *Tpbpa*, *Flt1*, *Prl8a8*, *Prl8a1*, and *Prl6a1*. Not all genes expressed from the spongiotrophoblast and glycogen cell lineage were reduced in expression coincident with the loss of this lineage. This is consistent with the known function of *Peg3* as a transcriptional repressor of placental hormone genes. The reciprocal relationship between the intensity of *Peg3* and *Prl3b1* signal in TGCs (**Figure 5**) provides further support for the role of *Peg3* as a transcriptional repressor for *Prl3b1* (Kim et al., 2013). *Psgs* are also targets for *Peg3* upregulated in response to loss of function of *Peg3* (Broad and Keverne, 2011) and possess multiple copies of the DNA-motif for *Peg3* (Lee et al., 2015). A key finding from this study is therefore that *Peg3* both positively and negatively regulates placental hormones through two independent and opposing mechanisms.

### Attenuated Response to Loss of *Peg3* in Female Placenta

Studies in mice are beginning to take into account the possibility of sexually dimorphic response of the placenta to genetic and environmental manipulations (Kalisch-Smith et al., 2017). Mutations involving the *Peg3* domain are known to have more severe effects on males than on females (Kim et al., 2012, 2013; Faisal et al., 2014). In comparison to male placenta, female *Peg3* KO placenta showed a more modest 37% loss of spongiotrophoblast, 29% loss of glycogen cells, and 33% reduction in placental glycogen. Aside from *Prl8a1* there were no significant reductions in the expression of genes found be reduced in male placenta, while *Psg17* and *Psg19*, co-expressed with *Prl8a8* in the spongiotrophoblast, were significantly elevated. *Prl8a8* was significantly different between male and female *Peg3* KO placenta but for most other criteria there were no significant differences between male and female mutants (**Supplementary Tables S1**–**S4**). These findings may be explained by naturally existing differences between male and female placenta. For example, *Tpbpa* was more highly expressed in WT male relative to WT female placenta and, although not significant with the numbers used in our study, the area of the Jz was slightly greater in WT male placenta. The sexually dimorphic response to loss of *Peg3* did not manifest as a significant difference in placental weight between male and female mutants or in fetal growth restriction, at least at E14.5. Fetal growth restriction has been reported at later time points and one study has recorded similar newborn weights between male and female mutant pups (He et al., 2016). However, this study examined an imprinting center mutation on a mixed genetic background and further work is required.

### Minimal Expression From the Maternal *Peg3* Allele

Expression of *Peg3* is higher in the placenta of E14.5 male B6/129-mixed background embryos compared to female (Faisal et al., 2014) but we did not find a significant difference in *Peg3* levels between male and female 129 placenta. We did find evidence for expression from the normally silenced maternal allele in both male and female placenta, with female placenta showing greater leaky expression. This expression represented <1% of the WT level, considerably lower than the normally active allele. While gene expression was analyzed only at E14.5 and we cannot exclude more significant *Peg3* expression from the maternal allele at earlier stages, it does seem unlikely that such a low level of expression would be sufficient to even partially rescue the spongiotrophoblast deficit. Sex-biased outcomes have been reported in response to mutations of YY1 binding sites in the *Peg3* promoter (He et al., 2017) suggesting another potential mechanism that may be tempering the phenotype in female placenta. Whether the mechanism is direct or indirect, these data highlight the important fact that female placenta compensate for the loss of *Peg3* far better than male placenta.

### Relevance to Prenatal Depression in Humans?

Our observation of a sexually dimorphic response in the placenta to loss of *Peg3* has potentially important implications. We recently reported reduced expression of the human *PEG3* gene in the placenta from women with either diagnosed depression in pregnancy or questionnaire determined depressive symptoms (Janssen et al., 2016). We observed the association in placenta from male infants but not female infants. We further observed a reduction in the expression of *human placental lactogen*, related to *Prls* genes in mice, again only in placenta from males. This current study highlights functional differences between male and female mouse placenta in response to loss of *Peg3*. This sexually dimorphic response might explain why we see a significant association between placental *PEG3* and prenatal depression in the placenta from boys and not girls in our human study. In light of these sex-specific phenotypes, it will be important to further explore both the behavioral phenotype in mice (McNamara et al., 2017) and gene expression data in human pregnancies.

## Summary

In conclusion, we report that loss of function of the imprinted *Peg3* gene results in the loss of both the spongiotrophoblast and glycogen cell lineages. This supports Haig’s original prediction that imprinted genes will be in conflict over the endocrine function of the placenta (Haig, 1996) and our hypothesis that this is achieved through the regulation of placental lineages that manufacture hormones (John and Hemberger, 2012; John, 2013, 2017). The sexually dimorphic response of the placenta to loss-of-function of *Peg3* and the remarkable fact that *Peg3* appears to be required both for the expression and repression of key placental hormones adds additional layers of complexity to studying the function of *Peg3* both in pregnancy and mammalian evolution.

## Materials and Methods

### Mouse Strains and Genotyping

Animal studies and breeding were approved by the Universities of Cardiff Ethical Committee and performed under a United Kingdom Home Office project license (RJ). All mice were housed under standard conditions throughout the study on a 12 h light–dark cycle with lights coming on at 07.00 h with a temperature range of 21°C ± 2 with free access to water (tap water), and standard chow. The *Peg3* targeted allele (Li et al., 1999) was crossed into the 129S2/SvHsd (Harlan, 129) strain and *Peg3* deficient fetuses were generated by crossing WT females with heterozygous *Peg^-/+^* (inherited targeted maternal allele; phenotypically wild type) males.

### Weighing Studies, *in situ* Hybridization, Histological Analyses, and LacZ staining

Fetal and placental wet weights were taken at the stated time point after a discernible plug, and normalized as previously described (Tunster et al., 2010). Genotyping and sex chromosome typing was obtained from yolk sac DNA as previously described (John et al., 2001), using primers 5′-TGCCACTCCCACTGTCCTTT and 5-GCACACAGCCTCTGCTCTGA for the *Peg3* targeted allele and 5′-TGGTCTGGACCCAAACGCTGTCCACA and 5′-GGCAGCAGCCATCACATAATCCAGATG for the X and Y chromosome *Ube1* gene. Placentas were fixed overnight in phosphate-buffered 4% paraformaldehyde, paraffin-embedded and 6 micron sections taken through the midline. Riboprobe preparation, *in situ* hybridization, H&E and PAS staining, and glycogen quantifications were performed as previously described (Tunster et al., 2010).

### Calculation of Area and Cell Number

Midline areas were calculated described (Tunster et al., 2010). Cell counts were performed on the same midline sections, using the “Cell Counter” plugin for ImageJ. *N* = 7 for each group (total *N* = 28).

### Quantitative RNA Analysis

RNA was extracted from whole placenta following careful removal of membranes and umbilicus. Quantitative PCR of reverse transcribed RNA (RT-qPCR) was performed and analyzed as described (Tunster et al., 2010). Data at https://osf.io/jhc83/.

### RNAscope

RNAscope was performed using RNAscope^®^ Multiplex Fluorescent Reagent Kit v2 (ACD) on FFPE placenta sections, following the manufacturer’s protocol with the following modifications and specifications. After deparaffinization and hydrogen peroxide incubation, slides were added to boiling RNAscope^®^ Target Retrieval for 15 min. Slides were incubated 15 min at 40°C in the HybEZ Oven with Protease Plus. To reduce the autofluorescence background, washes during the amplification steps with 1× Wash Buffer were increased from 2 × 2 min at RT to 3 x 5 min at RT. After amplification steps HRP-C1, HRP-C2, and HRP-C3 signals were developed. C1 corresponds to *Pcdh12* probe (ACD Mm-Pcdh12 489891) and TSA^®^Plus Cyanine 3 (Cy3), was assigned for this channel with a fluorophore concentration of 1:500. C2 corresponds to *Prl3b1* probe (ACD Mm-Prl3b1-C2 423671-C2) and TSA^®^Plus fluorescein was assigned for this channel with a fluorophore concentration of 1:5000. *Peg3* 88–994 (original probe Mm-Peg3-C3 492581), custom designed *Peg3* 3775–4659 (Mm-Peg3-C3 528201) were in Channel 3, and TSA^®^Plus Cyanine 5 (Cy5) was assigned for this channel with a fluorophore concentration of 1:1000. Under our RT-qPCR conditions *Prl3b1* peaks at cycle 15, *Peg3* peaks at cycle 21, and *Pcdh12* peaks at cycle 25 with a 854:48:1 relative ratio of gene expression (whole placenta) and fluorophores concentrations were adjusted with respect to mRNA levels for these genes at E14.5. Slides were counterstained with DAPI and mounted with Prolong Gold Antifade mounting medium.

### Imaging and Image Analysis

Whole placenta slides were scanned using Zeiss Axio Scan Z1. Higher resolution images were taken by the Confocal laser scanning microscope (LSM) 880 with Airyscan. Images were analyzed with ZEN 2 (blue edition) program. Fluorescence intensity 3D graphs (**Figure 5**) were generated with the image processing package Fiji with the interactive 3D service plot.

### Statistical Analyses

Statistical significance (probability values) was determined using ANOVA with *post hoc* analyses applying the Bonferroni correction.

## Author Contributions

ST performed the initial experiments, analyzed the data, and contributed to writing the manuscript. RB-S performed RNAscope work and PAS staining. GM collected and performed qPCR analysis on male andfemale samples. SH collected additional samples for glycogen analysis. RB-S and HC was involved in image processing and animal work. RJ and ST conceived and designed the experiments, interpreted the data, and wrote the paper.

## Conflict of Interest Statement

Since September 2017, GM has been employed by Frontiers. GM declared her affiliation with Frontiers. RJ is chief Specialty editor of Frontiers in Cell and Development Biology. The handling Editor states that the process nevertheless met the standards of a fair and objective review. The remaining authors declare that the research was conducted in the absence of any commercial or financial relationships that could be construed as a potential conflict of interest.
