# Supplementary material for: Peg3 Deficiency Results in Sexually Dimorphic Losses and Gains in the Normal Repertoire of Placental Hormones
Source: Front Cell Dev Biol. 2018 Sep 27;6:123. doi: 10.3389/fcell.2018.00123 (PMC6170603; doi:10.3389/fcell.2018.00123)
Supplement: Supplementary file 1 [file Table_1.DOCX]

**Table S1: Data for Figure 1**

|  | ANOVA  (F_3,81_ = ) | *Peg3*^+/-^ vs control | | Males v Females | |
| --- | --- | --- | --- | --- | --- |
|  |  | Males | Females | Control | *Peg3*^+/-^ |
| Fetal weight (mg) | 0.90  *p* = 0.444 | 217.4 mg ± 9.50  vs  207.0 mg ± 8.24  *p* = 0.491 | 216.1 mg ± 8.53  vs  201.1 mg ± 5.85  *p* = 0.366 | 217.4 mg ± 9.50  vs  216.1 mg ± 8.53  *p* = 0.911 | 207.0 mg ± 8.24  vs  201.1 mg ± 5.85  *p =* 0.254 |
| Placental weight (mg) | **9.48**  ***p* = 1.87 x 10^-5^** | **67.3 mg ± 2.48**  **vs**  **54.4 mg ± 2.46**  ***p* = 0.00141** | **69.8 mg ± 2.60**  **vs**  **57.3 mg ± 2.06**  ***p* = 0.00179** | 67.3 mg ± 2.48  vs  69.8 mg ± 2.60  *p* = 0.448 | 54.4 mg ± 2.46  vs  57.3 mg ± 2.06  *p =* 0.805 |
| F:P ratio | 4.03  *p* = 0.678 | 3.7 ± 0.20  vs  4.0 ± 0.23  *p* = 0.427 | 3.1 ± 0.12  vs  3.6 ± 0.14  *p* = 0.191 | 3.7 ± 0.20  vs  3.1 ± 0.12  *p* = 0.0681 | 4.0 ± 0.23  vs  3.6 ± 0.14  *p =* 0.500 |
| Glycogen (mg) | **5.88**  ***p* = 0.00110** | **0.21 mg ± 0.020 vs**  **0.12 mg ± 0.019**  ***p* = 0.00750** | **0.20 mg ± 0.020**  **vs**  **0.13 mg ± 0.016**  ***p* = 0.0389** | 0.21 mg ± 0.020  vs  0.20 mg ± 0.020  *p* = 0.653 | 0.12 mg ± 0.019  vs  0.13 mg ± 0.016  *p =* 0.203 |
| Glycogen (mg/g) | **3.13**  ***p* = 0.0303** | **3.16 mg/g ± 0.28**  **vs**  **2.05 mg/g ± 0.32**  ***p =* 0.0464** | 2.94 mg/g ± 0.25  vs  2.42 mg/g ± 0.26  *p =* 0.541 | 3.16 mg/g ± 0.28  vs  2.94 mg/g ± 0.25  *p =* 0.580 | 2.05 mg/g ± 0.32  vs  2.42 mg/g ± 0.26  *p =* 0.699 |

**Table S2: Data for Figure 2**

|  | ANOVA  (F_3,24_ = ) | *Peg3*^+/-^ vs control | | Males v Females | |
| --- | --- | --- | --- | --- | --- |
|  |  | Males | Females | Control | *Peg3*^+/-^ |
| Jz | **5.83**  ***p* = 0.00599** | **2.00 mm^2^ ± 0.20 vs**  **1.33 mm^2^ ± 0.15**  ***p* = 4.50 x 10^-9^** | **1.78 mm^2^ ± 0.13**  **vs**  **1.45 mm^2^ ± 0.07**  ***p* = 5.08 x 10^-8^** | 2.00 mm^2^ ± 0.20  vs  1.78 mm^2^ ± 0.13  *p* = 0.450 | 1.33 mm^2^ ± 0.15  vs  1.45 mm^2^ ± 0.07  *p* = 0.511 |
| Lz | 1.76  *p* = 0.183 | 6.30 mm^2^ ± 0.51  vs  5.44 mm^2^ ± 0.29  *p =* 0.353 | 5.33 mm^2^ ± 0.28  vs  5.43 mm^2^ ± 0.20  *p =* 0.637 | 6.30 mm^2^ ± 0.51  vs  5.33 mm^2^ ± 0.28  *p =* 0.330 | 5.44 mm^2^ ± 0.29  vs  5.43 mm^2^ ± 0.20  *p* = 0.993 |
| Total | 2.74  *p* = 0.066 | 8.30 mm^2^ ± 0.65  vs  6.77 mm^2^ ± 0.33  *p =* 0.108 | 7.11 mm^2^ ± 0.39  vs  6.88 mm^2^ ± 0.21  *p =* 0.429 | 8.30 mm^2^ ± 0.65  vs  7.11 mm^2^ ± 0.39  *p =* 0.234 | 6.77 mm^2^ ± 0.33  vs  6.88 mm^2^ ± 0.21  *p* = 0.858 |
| Lz:Jz | **3.78**  ***p* = 0.0237** | 3.29 ± 0.39  vs  4.10 ± 0.17  *p* = 0.150 | 3.03 ± 0.14  vs  3.80 ± 0.22  *p* = 0.157 | 3.29 ± 0.39  vs  3.03 ± 0.14  *p* = 0.470 | 4.10 ± 0.17  vs  3.80 ± 0.22  *p* = 0.796 |
| SpT | **10.3**  ***p* = 0.000113** | **699.8 ± 95.8**  **vs**  **360.7 ± 32.4**  ***p* = 9.06 x 10^-4^** | **671.5 ± 17.8**  **vs**  **425.1 ± 28.9**  ***p* = 0.00969** | 699.8 ± 95.8  vs  671.5 ± 17.8  *p* = 717 | 360.7 ± 32.4  vs  425.1 ± 28.9  *p* = 0.803 |
| GlyT | **7.12**  ***p* = 0.0014** | **1031.7 ± 95.9**  **vs**  **655.1 ± 54.6**  ***p* = 0.00654** | **937.8 ± 59.8**  **vs**  **664.7 ± 69.7**  ***p* = 0.0384** | 1031.7 ± 95.9  vs  937.8 ± 59.8  *p* = 0.727 | 655.1 ± 54.6  vs  664.7 ± 69.7  *p* = 0.926 |
| SpT:GlyT | 0.717  *p* = 0.552 | 0.66 ± 0.057  vs  0.58 ± 0.074  *p* = 0.958 | 0.74 ± 0.067  vs  0.72 ± 0.130  *p* = 0.853 | 0.66 ± 0.057  vs  0.74 ± 0.067  *p* = 0.578 | 0.58 ± 0.074  vs  0.72 ± 0.130  *p* = 0.291 |

**Table S3. Data for Figure 3**

|  |  | *Peg3*^+/-^ vs control | | Males vs Females | |
| --- | --- | --- | --- | --- | --- |
|  | ANOVA  (F_3,12_ = ) | Males | Females | Control | *Peg3*^+/-^ |
| *Tpbpa* | **4.52**  ***p* = 0.0243** | **0.69 ± 0.09**  ***p* = 0.0436** | 1.31 ± 0.15  *p* = 0.342 | **1.52 ± 0.22**  ***p* = 0.0468** | 0.80 ± 0.08  *p* = 0.399 |
| *Flt1* | 2.64  *p* = 0.0974 | 0.68 ± 0.08  *p* = 0.144 | 1.17 ± 0.21 *p* = 0.872 | 1.27 ± 0.18  *p* = 0.417 | 0.74 ± 0.12  *p* = 0.386 |
| *Prl8a8* | **7.12**  ***p* = 0.0053** | **0.60 ± 0.04**  ***p* = 0.00639** | 1.20 ± 0.17 *p* = 0.273 | 1.32 ± 0.16  *p* = 0.122 | **0.66 ± 0.07**  ***p* = 0.0251** |
| *Prl3c1* | 0.973  *p* = 0.438 | 1.34 ± 0.37  *p* = 0.392 | 1.55 ± 0.53 *p* = 0.146 | 1.24 ± 0.37  *p* = 0.159 | 1.08 ± 0.35  *p* =0.785 |
| *Prl7a2* | 0.372  *p* = 0.775 | 1.07 ± 0.35  *p* = 0.586 | 1.06 ± 0.16 *p* = 0.802 | 0.84 ± 0.11  *p* = 0.471 | 0.85 ± 0.28  *p* = 0.595 |
| *Prl3b1* | 1.33  *p* = 0.310 | 0.76 ± 0.09  *p* = 0.604 | 1.12 ± 0.28 *p* = 0.917 | 1.14 ± 0.15  *p* = 0.503 | 0.78 ± 0.11  *p* = 0.651 |
| *Prl8a1* | **8.64**  ***p* = 0.0025** | **0.47 ± 0.08**  ***p* = 0.0127** | **0.49 ± 0.08 *p* = 0.0223** | 1.07 ± 0.21  *p* = 0.267 | 1.02 ± 0.14  *p* = 0.950 |
| *Prl8a6* | 0.527  *p* = 0.627 | 1.25 ± 0.35  *p* = 0.376 | 1.35 ± 0.66 *p* = 0.889 | 0.77 ± 0.24  *p* = 0.897 | 0.71 ± 0.33  *p* = 0.137 |
| *Prl8a9* | 2.44  *p* = 0.114 | 1.30 ± 0.22  *p* = 0.820 | 1.49 ± 0.26 *p* = 0.223 | 0.99 ± 0.12  *p* = 0.960 | 0.86 ± 0.18  *p* = 0.718 |
| *Psg17* | **3.90**  ***p* = 0.0372** | 1.29 ± 0.20  *p* = 0.782 | **1.73 ± 0.35 *p* = 0.0410** | 1.00 ± 0.15  *p* = 0.990 | 0.75 ± 0.15  *p* = 0.402 |
| *Psg18* | 0.732  *p* = 0.553 | 1.08 ± 0.20  *p* = 0.716 | 1.34 ± 0.27 *p* = 0.793 | 1.13 ± 0.17  *p* = 0.632 | 0.91 ± 0.21  *p* = 0.226 |
| *Psg19* | **3.77**  ***p* = 0.0485** | 1.32 ± 0.23  *p* = 0.466 | **1.57 ± 0.23 *p* = 0.0145** | 1.03 ± 0.09  *p* = 0.877 | 0.87 ± 0.18  *p* = 0.767 |
| *Psg21* | 2.17  *p* = 0.145 | 1.31 ± 0.24  *p* = 0.553 | 1.51 ± 0.30 *p* = 0.354 | 1.13 ± 0.22  *p* = 0.192 | 0.98 ± 0.19  *p* = 0.915 |
| *Pcdh12* | **4.94**  ***p* = 0.0185** | 0.91 ± 0.15; *p* = 0.549 | 0.87 ± 0.15 *p* = 0.785 | **1.56 ± 0.19**  ***p* = 0.0116** | 1.63 ± 0.34  *p* = 0.0885 |
| *Gjb3* | **3.00**  ***p* = 0.0323** | 1.15 ± 0.20  *p* = 0.679 | 0.97 ± 0.11 *p* = 0.892 | 1.28 ± 0.08  *p* = 0.416 | 1.51 ± 0.30  *p* = 0.156 |
| *Prl7b1* | 1.58  *p* = 0.245 | 0.75 ± 0.23  *p* = 0.294 | 0.74 ± 0.15 *p* = 0.733 | 1.35 ± 0.36  *p* = 0.206 | 1.37 ± 0.35  *p* = 0.366 |
| *Prl6a1* | **4.10**  ***p* = 0.0323** | **0.59 ± 0.11**  ***p* = 0.0456** | 0.85 ± 0.20 *p* = 0.848 | 1.58 ± 0.28  *p* = 0.0973 | 1.09 ± 0.27  *p* = 0.801 |
| *Prl2a1* | **1.79**  ***p* = 0.0203** | 0.80 ± 0.16  *p* = 0.124 | 1.12 ± 0.45 *p* = 0.768 | **1.80 ± 0.41**  ***p* = 0.0346** | 1.30 ± 0.50  *p* = 0.798 |
| *Gbe1* | **5.86**  ***p* = 0.0105** | 0.84 ± 0.08  *p* = 0.336 | 1.04 ± 0.17 *p* = 0.760 | **1.54 ± 0.24**  ***p* = 0.0178** | 1.24 ± 0.12  *p* = 0.236 |
| *Gyg* | 1.03  *p* = 0.412 | 1.05 ± 0.13  *p* = 0.749 | 1.27 ± 0.18 *p* = 0.673 | 1.14 ± 0.11  *p* = 0.622 | 0.94 ± 0.15  *p* = 0.219 |
| *Gys* | 1.35  *p* = 0.306 | 0.98 ± 0.05  *p* = 0.738 | 1.11 ± 0.07 *p* = 0.454 | 1.08 ± 0.08  *p* = 0.920 | 0.95 ± 0.02  *p* = 0.995 |
| *Ugp2* | **14.0**  ***p* = 0.00301** | 0.87 ± 0.08  *p* = 0.348 | 0.97 ± 0.17 *p* = 0.858 | **1.84 ± 0.25**  ***p* = 0.00132** | **1.65 ± 0.23**  ***p* = 0.00951** |
| *Cdkn1c* | **3.544**  ***p* = 0.0480** | 0.98 ± 0.26  *p* = 0.908 | 0.91 ± 0.20 *p* = 0.633 | **1.83 ± 0.37**  ***p* = 0.0180** | 1.96 ± 0.55  *p* =0.181 |
| *Hand1* | 0.531  *p* = 0.670 | 1.16 ± 0.13  *p* = 0.416 | 1.00 ± 0.13 *p* = 0.917 | 0.91 ± 0.12  *p* = 0.269 | 1.06 ± 0.11  *p* =0.856 |
| *Tle3* | **10.7**  ***p* = 0.00123** | 0.88 ± 0.16  *p* = 0.808 | 0.92 ± 0.07 *p* = 0.431 | **0.64 ± 0.08**  ***p* = 0.00858** | **0.61 ± 0.09**  ***p* = 0.00980** |
| *Ctsq* | 0.181  *p* = 0.908 | 0.97 ± 0.28  *p* = 0.931 | 1.33 ± 0.73 *p* = 0.922 | 1.52 ± 0.71  *p* = 0.121 | 0.92 ± 0.25  *p* = 0.301 |
| *Prl2c* | 0.294  *p* = 0.829 | 0.91 ± 0.15  *p* = 0.398 | 1.03 ± 0.17 *p* = 0.715 | 0.97 ± 0.09  *p* = 0.870 | 0.86 ± 0.18  *p* = 0.347 |
| *Flk1* | 1.50  *p* = 0.266 | 0.94 ± 0.14  *p* = 0.693 | 1.13 ± 0.24 *p* = 0.991 | 1.41 ± 0.16  *p* = 0.478 | 1.17 ± 0.27  *p* = 0.165 |
| *Dlx3* | 1.74  *p* = 0.211 | 0.84 ± 0.12  *p* = 0.635 | 0.94 ± 0.12 *p* = 0.638 | 0.87 ± 0.14  *p* = 0.274 | 0.78 ± 0.08  *p* = 0.584 |
| *Tfeb* | 2.39  *p* = 0.120 | 0.91 ± 0.25  *p* = 0.526 | 0.65 ± 0.13 *p* = 0.256 | 0.68 ± 0.16  *p* = 0.309 | 0.95 ± 0.24  *p* = 0.841 |
| *Syna* | 2.21  *p* = 0.140 | 1.15 ± 0.1  *p* = 0.868 | 0.98 ± 0.13 *p* = 0.918 | 1.10 ± 0.14  *p* = 0.811 | 1.29 ± 0.13  *p* = 0.254 |
| *Ly6e* | 0.807  *p* = 0.514 | 1.01 ± 0.14  *p* = 0.939 | 0.96 ± 0.12 *p* = 0.425 | 0.85 ± 0.13  *p* = 0.321 | 0.90 ± 0.09  *p* = 0.255 |
| *Gcm1* | 0.561  *p* = 0.651 | 1.05 ± 0.2  *p* = 0.814 | 1.10 ± 0.11 *p* = 0.817 | 1.17 ± 0.23  *p* = 0.770 | 1.12 ± 0.12  *p* = 0.502 |
| *Synb* | 0.517  *p* = 0.679 | 1.14 ± 0.13  *p* = 0.682 | 0.97 ± 0.12 *p* = 0.612 | 0.89 ± 0.11  *p* = 0.654 | 1.04 ± 0.11  *p* = 0.157 |
| *Cebpa* | 1.37  *p* = 0.300 | 1.20 ± 0.24  *p* = 0.129 | 0.94 ± 0.25 *p* = 0.792 | 0.63 ± 0.17  *p* = 0.595 | 0.79 ± 0.16  *p* =0.119 |

**Table S4. Data for Figure 6**

| *Peg3*^+/-^ vs control | | | |
| --- | --- | --- | --- |
|  | ANOVA  (F_3,12_ = ) | Males | Females |
| *Peg3* | **163.5**  ***p* = 5.39 x 10^-10^** | **0.00157**  **± 0.00070**  ***p* = 4.50 x 10^-9^** | **0.00599 ± 0.00139**  ***p* = 5.08 x 10^-8^** |
| Females vs Males | | | |
|  | ANOVA  (F_3,12_ = ) | Control (F) vs control (M) | *Peg3*^+/-^ (F) vs *Peg3*^+/-^ (M) |
| *Peg3* | **163.5**  ***p* = 5.39 x 10^-10^** | 1.38 ± 0.40  *p* = 0.251 | **5.19 ± 2.18**  ***p* = 9.9 x 10^-4^** |
