# Supplementary figures and images for: Peg3 Deficiency Results in Sexually Dimorphic Losses and Gains in the Normal Repertoire of Placental Hormones
Source: Front Cell Dev Biol. 2018 Sep 27;6:123. doi: 10.3389/fcell.2018.00123 (PMC6170603; doi:10.3389/fcell.2018.00123)

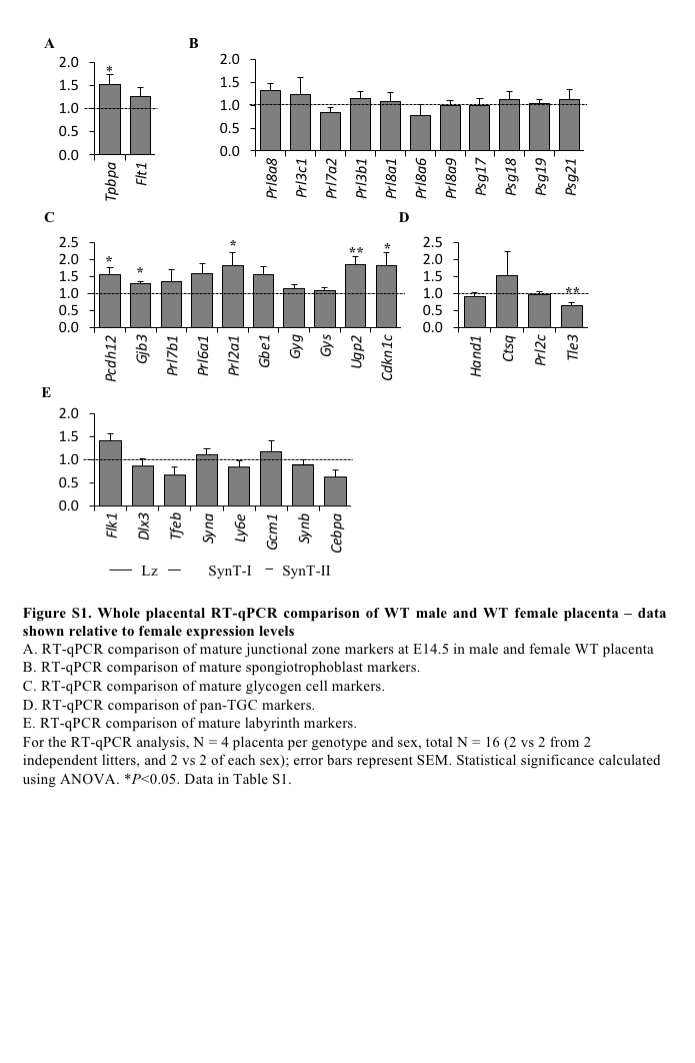

Supplement: Supplementary file 2 [file Image_1.TIFF]

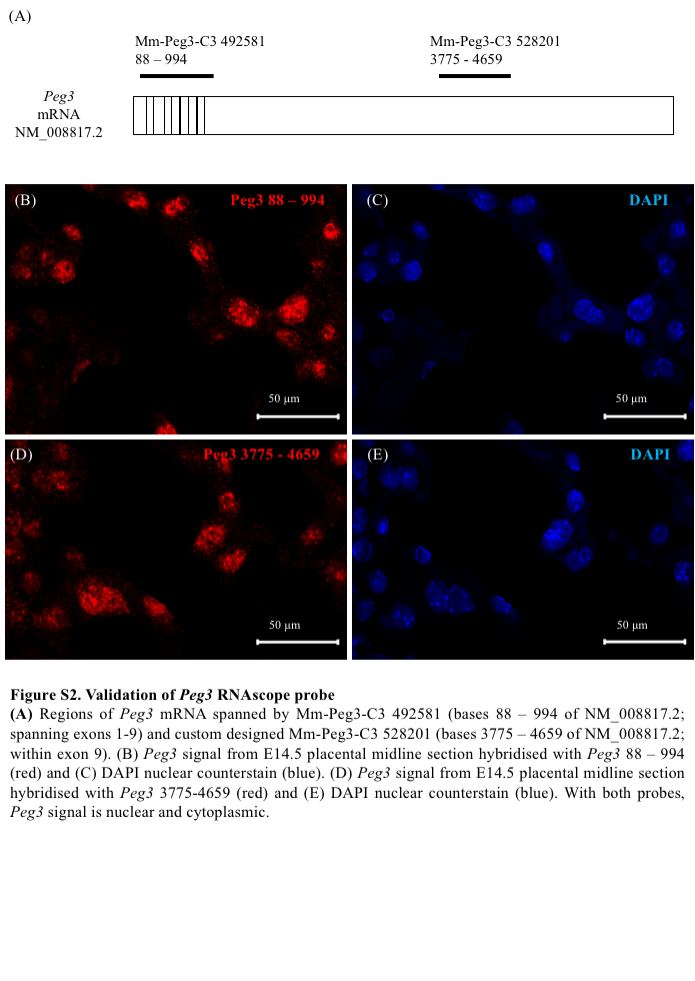

Supplement: Supplementary file 3 [file Image_2.TIFF]

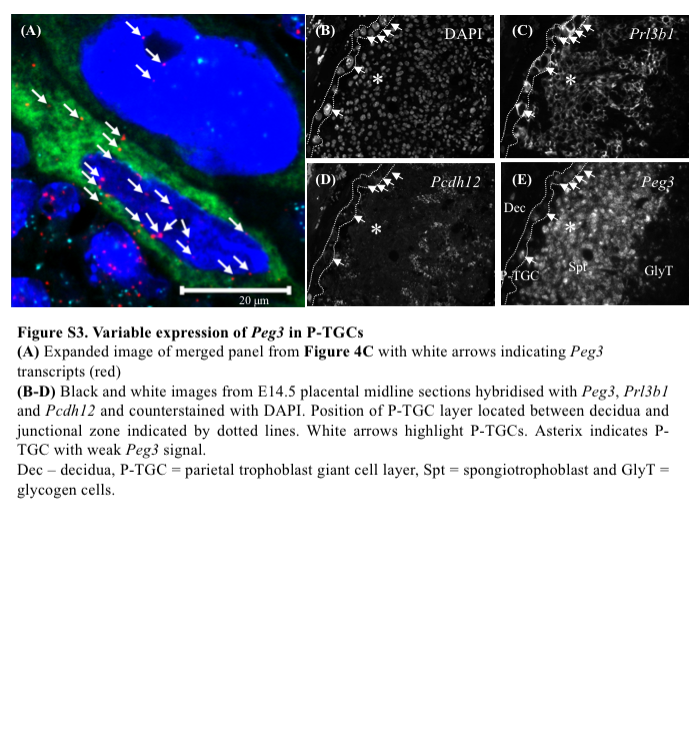

Supplement: Supplementary file 4 [file Image_3.TIFF]
